# Arterial oxygen pressure during veno-venous extracorporeal membrane oxygenation may be increased by advancing the tip of the drainage cannula into the superior vena cava: a case report

**DOI:** 10.1007/s10047-024-01448-w

**Published:** 2024-05-21

**Authors:** Tomoyuki Nakamura, Naohide Kuriyama, Yoshitaka Hara, Hidefumi Komura, Naoki Hoshino, Soshi Miyamoto, Ken Sawada, Takahiro Kawaji, Satoshi Komatsu, Osamu Nishida

**Affiliations:** 1 Department of Anesthesiology and Critical Care Medicine, Fujita Health University School of Medicine, 1-98 Dengakugakubo, Kutsukake-cho, Toyoake, Aichi 470-1192 Japan; 2 Department of Cardiology, Fujita Health University School of Medicine, Toyoake, Japan

**Keywords:** Venovenous extracorporeal membrane oxygenation (V–V ECMO), Drainage cannula, Reinfusion cannula, Superior vena cava, Recirculation

## Abstract

A simple and robust method for veno-venous extracorporeal membrane oxygenation (V–V ECMO) involves a drainage cannula into the inferior vena cava via the femoral vein (FV) and a reinfusion cannula into the right atrium (RA) via the internal jugular vein (IJV) (F–J configuration). However, with this method, the arterial oxygen (PaO_2_) is said to remain below 100 mmHg.

Since recently, in our ICU, to prevent drainage failure, we apply a modification from the commonly practiced F–J configuration by advancing the tip of the drainage cannula inserted via the FV into the superior vena cava (SVC) and crossing the reinfusion cannula inserted via the IJV in the RA (F(SVC)–J(RA) configuration). We experienced that this modification can be associated with unexpectedly high PaO_2_ values, which here we investigated in detail.

Veno-arteriovenous ECMO was induced in a 65-year-old male patient who suffered from repeated cardiac arrest due to acute respiratory distress syndrome. His chest X-ray images showed white-out after lung rest setting, consistent with near-absence of self-lung ventilation. Cardiac function recovered and the system was converted to F(SVC)–J(RA) configuration, after which both PaO_2_ and partial pressure of pulmonary arterial oxygen values remained high above 200 mmHg. Transesophageal echocardiography could not detect right-to-left shunt, and more efficient drainage of the native venous return flow compared to common F–J configuration may explain the increased PaO_2_.

Although the F(SVC)-J(RA) configuration is a small modification of the F–J configuration, it seems to provide a revolutionary improvement in the ECMO field by combining robustness/simplicity with high PaO_2_ values.

**Supplementary Information:**

The online version contains supplementary material available at 10.1007/s10047-024-01448-w.

## Introduction

Veno-venous extracorporeal membrane oxygenation (V–V ECMO) can be broadly classified into four strategies depending on the cannulation configuration: (1) a drainage cannula into the inferior vena cava (IVC) via the femoral vein (FV) and a reinfusion cannula into the right atrium (RA) via the internal jugular vein (IJV) (F–J configuration); (2) a drainage cannula into the RA via the IJV and a reinfusion cannula into the IVC via the FV (J–F configuration); (3) a drainage cannula into the IVC via the FV and a reinfusion cannula into the RA via the opposite side of the FV (F-F configuration); and, (4) dual lumen cannula (DLC) via the IJV, whose drainage port open to the superior vena cava (SVC) and the IVC, and reinfusion port toward the RA (DLC configuration).

During V–V ECMO, the systemic blood flow becomes a mixture of the oxygenated reinfusion ECMO flow and the deoxygenated native venous return flow from the whole body that did not pass through the ECMO circuit. Furthermore, related to the positions of the cannulae, some recirculation—the return of extracorporeal oxygenated blood into the V–V ECMO system without having passed through the systemic circulation—is inevitable, and this also reduces the levels of blood oxygenation that can be achieved [1, 2]. It is generally understood that if V–V ECMO is used in the absence of functional self-lung ventilation, the partial pressure of arterial oxygen (PaO_2_) cannot achieve normal levels and will remain below 100 mmHg [1, 2], which is typically associated with oxygen saturation in arterial blood (SaO_2_) reaching only 60–90% [2]. Therefore, special management, such as by increasing hemoglobin levels, to tolerate low SaO_2_ for maintaining a sufficiently high arterial oxygen content despite low SaO_2_, may be required.

In our ICU, for performing V-V ECMO, we came to prefer an F–J configuration because it is less likely to cause a substantial recirculation. To prevent drainage failure, we have modified this method by advancing the tip of the drainage cannula into the SVC, which results in the reinfusion cannula crossing the drainage cannula in the RA; we refer to this configuration is as “(F(SVC)–J(RA) configuration” and, according to Extracorporeal Life Support Organization nomenclature rules, this type of ECMO can be named “V_f svc_–V_j a_ ECMO” [3]. Having used this F(SVC)–J(RA) configuration in tens of cases already, although without detailed investigations of various relevant aspects, we multiple times experienced unexpectedly high PaO_2_ values, with the variation seemingly related to factors that we still need to investigate (unpublished data). Higher PaO_2_ values during V–V ECMO are desirable, and therefore we investigated one case in detail by for example also using transesophageal echocardiography (TEE) and a pulmonary arterial catheter (PAC), and this case is described here. Using V–V ECMO with F(SVC)–J(RA) configuration in a 65-year-old male patient suffering from severe respiratory failure, his PaO_2_ and partial pressure of pulmonary arterial oxygen (P_PA_O_2_) were elevated far above 200 mmHg, despite lung rest setting and the near-absence of self-lung ventilation. We discuss the possible mechanism by which the F(SVC)–J(RA) configuration may be associated with such high PaO_2_.

## Case presentation

The patient was a 65-year-old male (height 166 cm, body weight 66 kg, body surface area 1.7 m^2^), who after total cystectomy for bladder cancer was treated by adjuvant nivolumab therapy. After 11 courses of the therapy, the patient developed systemic dermatitis as an immune-related adverse event and was hospitalized. On day 16 of the hospitalization, he was admitted to the intensive care unit (ICU) for post-cardiac arrest resuscitation, because of an acute respiratory distress syndrome triggered by aspiration pneumonia.

Upon admission to the ICU, he received pressure-controlled ventilation, with a positive end expiratory pressure of 10 cm H_2_O, a peak inspiratory pressure of 25 cm H_2_O, a respiratory rate of 30/min, and the fraction of inspiratory oxygen (F_I_O_2_) set at 1.0. Other blood values were: arterial blood gas pH, 7.032; partial pressure of carbon dioxide (PaCO_2_), 86.2 mmHg; PaO_2_, 56.9 mmHg; base excess, − 7.9; and lactic acid, 7.1 mmol/L. His ventilation had drastically deteriorated and he produced frothy sputum, and for aiding him with manual ventilation using the Jackson-Reese circuit a pressure of 40 cm H_2_O or higher was required. Veno-arterial (V-A) ECMO was introduced (Cardiohelp HLS system^™^ (Getinge, Sweden)) because of the patient’s repeated cardiac arrest due to hypoxia. For drainage, a venous HLS cannula^™^ 25Fr 55 cm (Getinge, Sweden) was inserted from the right FV and the tip of it was advanced to the SVC through the IVC and RA, and an arterial HLS cannula^™^ 17Fr 15 cm was inserted into the right femoral artery for reinfusion. One hour later, an additional reinfusion cannula (arterial HLS cannula^™^ 23Fr 23 cm) was inserted from the right IJV and the configuration was converted to veno-arteriovenous (V–AV) ECMO. The positions of the drainage and reinfusion cannulae were adjusted under fluoroscopic guidance, and contrast was used to help choose the exact positions where recirculation was smallest.

**Fig. 1:**
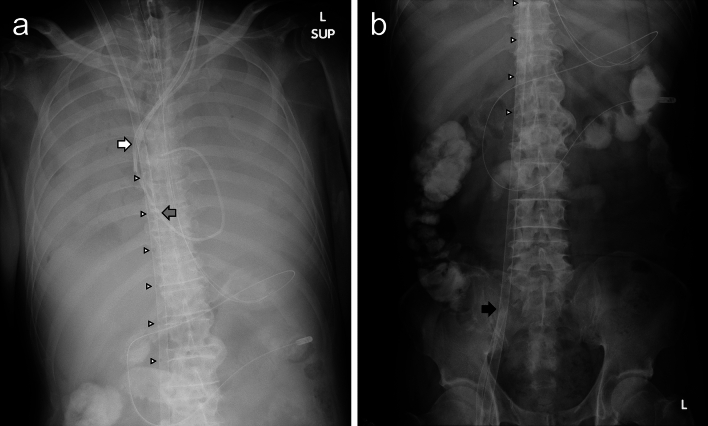
X-ray images after induction of veno-arteriovenous (V–AV) ECMO and lung rest ventilator setting. White arrow: the tip of the drainage cannula. Gray arrow: the tip of the venous reinfusion cannula. Black arrow: the tip of the arterial reinfusion cannula. White triangles: side holes of the drainage cannula. **a** Chest X-ray image. A venous reinfusion cannula and a central venous catheter were inserted from the right internal jugular vein. A pulmonary artery catheter and a double lumen catheter for blood purification were inserted from the left internal jugular vein. **b** Abdominal X-ray image. A drainage cannula was inserted from the right femoral vein and an arterial reinfusion cannula was inserted from the right femoral artery

Then a PAC was inserted into the right pulmonary artery via the left IJV. Continuous muscle relaxation was initiated and the chest X-ray image was white-out (Fig. 1a) under lung rest setting. The minute volume (MV) was about 0.10 L/min, confirming a near-absence of lung-ventilation. P_PA_O_2_ sampling from the tip of the PAC showed 300.6 mmHg, while, at the same time, PaO_2_ sampling from the left radial artery showed 304.8 mmHg. After hypoxia had improved and cardiac function recovered, the reinfusion cannula in the right femoral artery was removed 21 h after ECMO induction, and the configuration was converted to V–V ECMO. Fluoroscopic guidance showed that the reinfusion ECMO flow directly entered into the IVC (Supplemental Video 1), so the position of the tip of the reinfusion cannula was adjusted by pulling it upward to the upper RA (Supplemental Video 2). This time, although there still was almost no lung-ventilation with an MV of 0.10–0.15 L/min, the oxygen values P_PA_O_2_ 296.3 mmHg and PaO_2_ 268.4 mmHg were measured. Table 1 summarizes the settings of V-AV ECMO, V-V ECMO, and lung rest ventilator used during the procedure.

**Table 1:** The settings during ECMO management

V–AV ECMO setting (ECMO run: 1–21 h) ECMO flow: 4.0 L/min (pump speed: 2590 rpm) Arterial reinfusion: 1.0–1.5 L/min / Venous reinfusion: 3.0–3.5 L/min F_D_O_2_: 0.80–1.0 Sweep gas flow: 3.0 L/min
V-V ECMO setting (ECMO run: 21–64 h) ECMO flow: 4.0 L/min (pump speed: 2630 rpm) F_D_O_2_: 0.70–1.0 Sweep gas flow: 3.0 L/min
Lung rest ventilator setting (ECMO run: 1–64 h) PCV F_I_O_2_: 0.30 PEEP: 5 cm H_2_O PIP: 10 cm H_2_O Mandatory respiratory rate: 5 breaths/min

*ECMO* extracorporeal membrane oxygenation, *V-AV* veno-arteriovenous, *V-V* veno-venous, *F*_*D*_*O*_*2*_ fraction of delivered oxygen, *PCV* pressure control ventilation, *F*_*I*_*O*_*2*_ fraction of inspiratory oxygen, *PEEP* positive end expiratory pressure, *PIP* peak inspiratory pressure

TEE was performed 45 h after starting ECMO to investigate the possible presence of a right-to-left shunt. No retrograde blood flow was seen on color Doppler images at the coronary artery inlet of the sinus Valsalva. The atrial septum appeared to have a slit-like gap on tomography images, but there was no clear right-to-left shunt flow on color Doppler images. The reinfusion ECMO flow was directed to the right ventricle, and during systole it was repelled by the closed tricuspid valve and directed toward the atrial septum, but the color flow did not reach the left atrium. A microbubble test did not detect bubble inflow into the left atrium, which suggests the absence of a right-to-left shunt flow (Supplemental Video 3).

When TEE was performed, we set F_D_O_2_ to 1.0 and evaluated the oxygen saturation and recirculation ratio. The oxygen saturation in the left innominate venous blood was 80.9%, the oxygen saturation in the RA from PAC central venous pressure port was 88.3%, the oxygen saturation before membrane was 84.7%, and the oxygen saturation after membrane was 99.9%. The recirculation ratio calculated by using the oxygen saturation in the left innominate venous blood, representing the saturation of venous blood returning to the vena cavae just before being drained by the ECMO circuit, was 20.0%.

**Fig. 2:**
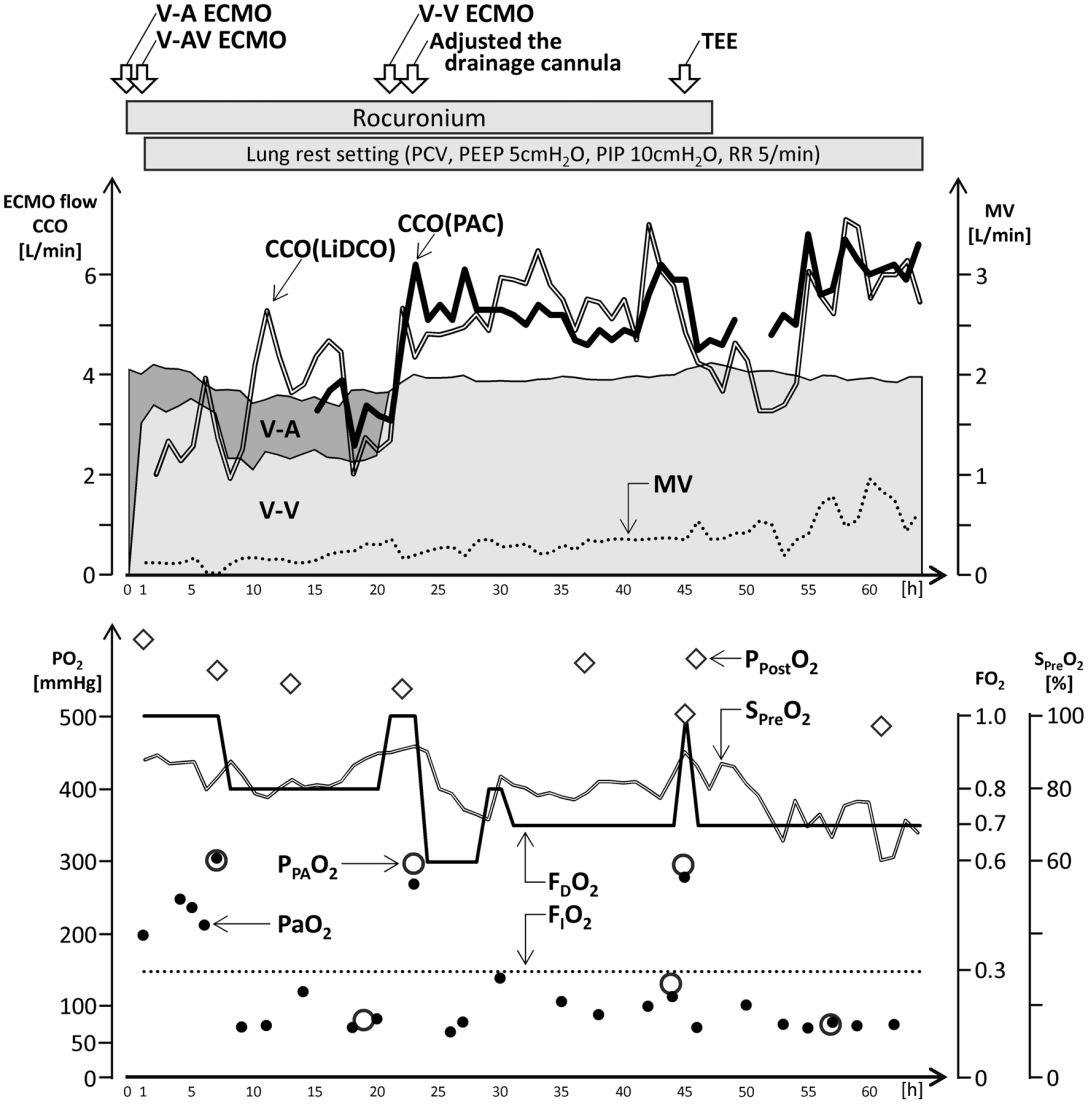
Clinical course and treatment schedule within the 64 h under lung rest setting after introduction of ECMO in a 65-year-old patient. *ECMO,* extracorporeal membrane oxygenation; *V-A,* veno-arterial; *V-AV,* veno-arteriovenous; *V-V,* veno-venous; *TEE*, transesophageal echocardiography; *PCV,* pressure control ventilation; *PEEP*, positive end expiratory pressure; *PIP*, peak inspiratory pressure; Rocuronium, a muscle relaxant; *RR*, respiratory rate; *CCO*, continuous cardiac output; *CCO(PAC)*, continuous cardiac output measured by pulmonary artery catheter; *CCO(LiDCO),* continuous cardiac output measured by LiDCO; *MV*, minute volume; *PO*_*2*_, partial pressure of oxygen; *P*_*PA*_*O*_*2*_, partial pressure of pulmonary artery oxygen; *PaO*_*2*_, partial pressure of arterial oxygen; *FO*_*2*_, fraction of oxygen; *F*_*D*_*O*_*2*_, fraction of delivered oxygen; *F*_*I*_*O*_*2*_, fraction of inspiratory oxygen; *P*_*Post*_*O*_*2*_, partial pressure of oxygen after membrane; *S*_*Pre*_*O*_*2*_, oxygen saturation before membrane

Continuous cardiac output measured by Vigilance II^™^ (Edwards Lifesciences, United States) or by LiDCO rapid V3^™^ (LiDCO Ltd., United Kingdom) was 5.0–6.0 L/min, while the ECMO flow was about 4.0 L/min and the MV under lung rest setting was below 1.0 L/min. However, the systemic oxygenation was maintained, so the target was set at PaO_2_ 80 mmHg and PaCO_2_ 40 mmHg, the fraction of delivered oxygen of ECMO (F_D_O_2_) was set at 0.70, and the sweep gas flow was set at 3.0 L/min. The MV was below 1.0 L/min, and the systemic oxygenation was maintained for 18 h after the stop of continuous muscle relaxation (Fig. 2).

On day 12 of the ICU admission, the ECMO was weaned off. On day 13, the ventilation was weaned off and the patient was extubated. Contrast-enhanced computed tomography on day 14 showed thrombus from the right common iliac vein to the FV, but not from the SVC to the bilateral IJVs. On day 24, the patient had fully recovered and was discharged from the ICU.

## Discussion

In the case described here, PaO_2_ was much higher during V-V ECMO than common during ECMO management. Possible physiological explanations for this may involve (1) little or no ECMO recirculation, (2) the presence of a right–to-left shunt, and/or (3) remaining effective self-lung ventilation.

The extent of recirculation in V–V ECMO is determined by the method of drainage and reinfusion, as well as by the positioning and shapes of the of the drainage and reinfusion cannulae. For the F–J configuration, it is recommended that the tip of the drainage cannula is positioned near the RA–IVC junction, with the side holes located within the intrahepatic inferior vena cava where vascular collapse is less likely to occur [1]. However, this can also result in drainage failure and, to avoid this, the tip of the drainage cannula is often advanced into the RA. In our experience, advancing the tip of the drainage cannula through the RA and deeper into the SVC is the least likely to cause drainage failure. Venous blood drainage in V–A ECMO is more efficient when the tip of the drainage cannula is positioned higher in the SVC as compared to a lower position [4], and the same may be true in V–V ECMO.

It has been described that the drainage cannula is draining blood mainly through a few proximal side holes and only very little through the tip unless these side holes are occluded [5, 6]. However, this was an ex vivo assessment in still water, and an upper body venous return flow to the RA as must happen in vivo was not considered. In the F(SVC)–J(RA) configuration, the drainage cannula directly receives the upper body venous return flow that is on its way to the RA. Therefore, even without negative pressure at the tip of the drainage cannula, the upper body venous return flow would have spontaneously flowed into the drainage cannula from the tip and bypassed the RA through the drainage cannula. On the other hand, in the F(SVC)–J(RA) configuration in this case study, the most proximal side hole was located at the level of the renal vein (Fig. 1b). Therefore, the native venous return flow might be efficiently drained from both the SVC and the IVC, as if being drained by two drainage cannulae. It is recommended that the drainage cannula and the reinfusion cannula should be placed separately by about 10 cm to minimize recirculation in configurations with two-cannulae accesses such as F–J, J–F, and F–F configurations [1, 7, 8], but this does not apply to the F(SVC)–J(RA) configuration. Through the side holes open in the RA, little or no drainage may occur, because they are fifth from the proximal side and the negative pressure may be little or none, and the reinfusion flow did not stream toward these side holes of the drainage cannula directly. Therefore, in the RA, the entering native venous return flow was minimal and the percentage of the reinfusion ECMO flow was higher.

Bonacchi et al. reported on an “χ configuration,” in which the reinfusion cannula was angled so that the reinfusion flow was directed to the tricuspid valve and the drainage cannula was advanced in the RA just below the SVC [9]. However, in the 16 cases presented in that study, PaO_2_ was 97.8 ± 12.8 mmHg, and no case showed similar high PaO_2_ values as described in our present case report. Bonacchi et al. emphasized the shape and the tip direction of the reinfusion cannula, but we believe that the drainage cannula is more important than the reinfusion cannula. Bonacchi et al. used a drainage cannula with multiple side holes, but no specific product name was given. Differences in the shape, number, spacing, and size of the side holes of the drainage cannula may have contributed to creating the different results between our studies. Importantly, in the Bonacchi et al. study, the tip of the drainage cannula was not advanced into the SVC, and this spatially slight difference may have had a significant impact.

If a right-to-left shunt of the ECMO flow is present, high PaO_2_ may be achieved. Possibilities for right-to-left shunt include patent foramen ovale and coronary regurgitation. Patent foramen ovale is asymptomatic in 20–25% of patients [10]. Since in our case the tip of the reinfusion cannula was located close to the coronary sinus, the possibility of part of the ECMO flow going back through the coronary sinus into the coronary artery cannot be ruled out. However, clear indications for right-to-left shunt were not found, as TEE with bubble test did not detect any flow indicative of regurgitation or bubbles in the left atrium or sinus of Valsalva deriving from the reinfusion ECMO flow.

If air remains in the lung field even during lung rest, it cannot be ruled out that the very low self-lung ventilation and apneic oxygenation [11] may nevertheless produce a high PaO_2_. However, in the here presented case, the F_I_O_2_ under lung rest setting was 0.30, making such scenario unlikely. Furthermore, the chest X-ray imaging showed a complete “white-out” with no air in the lung field and the MV was less than the normal tidal volume. Therefore, a hypothesis that the observed high PaO_2_ derived from a very low self-lung ventilation or apneic oxygenation should probably be negated.

The DLC configuration is considered the most effective method to reduce recirculation [1, 2], and the Avalon Elite^™^ Bi-Caval Dual Lumen Catheter (Getinge, Sweden) has been available in Japan since 2018. Avalon Elite^™^ has several advantages such as a reduced recirculation and easier rehabilitation. However, it also has many disadvantages, such as high cost and difficulties in adjusting the optimal cannula position and in sustaining a sufficient ECMO flow, as well as the possibility of serious complications such as cardiovascular perforation. Difficulties in sustaining a sufficient flow when using Avalon Elite^™^ relate to the actual size of the drainage port being small due to the double lumen structure; according to pressure drop diagram analysis [12], the drainage performance of Avalon Elite^™^ 31Fr is equivalent to venous HLS cannula^™^ 23Fr 38 cm and Avalon Elite^™^ 27Fr is equivalent to venous HLS cannula^™^ 21Fr 55 cm.

In Europa and the United States, ProtekDUO^™^ and Spectrum Medical’s dual lumen cannula systems are available, and in these the drainage port is open to the RA and the reinfusion port is open to the pulmonary artery so that the right ventricle is bypassed [veno-pulmonary artery ECMO (V–P ECMO)] [13, 14]. In V–P ECMO, recirculation should theoretically be 0% and it can provide support for right heart failure by acting as a percutaneous right ventricular assist device. ProtekDUO^™^ has a lineup of 31Fr and 29Fr and Spectrum Medical’s dual lumen cannula has a lineup of 31Fr, 27Fr and 24Fr for adult use. However, the actual effective size of the drainage port is even smaller because the reinfusion port is positioned in the center of the lumen of the cannula [14, 15]. As with the Avalon Elite^™^, sufficient ECMO flow may not be achieved, and a veno-venopulmonary (V–VP) configuration with the addition of another large drainage cannula has been proposed [16]. Although our method, the F(SVC)–J(RA) configuration, cannot provide support for right heart failure, it is an excellent method that can maintain a PaO_2_ as high as that of a DLC configuration while providing a sufficient ECMO flow, simply by adjusting the position of the tip of the drainage cannula.

There are several limitations to this report. Firstly, the effect of the F(SVC)–J(RA) configuration cannot be well determined from PaO_2_ during V–AV ECMO, as the PaO_2_ may also reflect the retrograde arterial ECMO flow via the right femoral artery and the arterial line was in the left radial artery. However, even after converting the configuration to V-V ECMO, the same high PaO_2_ was achieved. Therefore, also during V-AV ECMO, the high PaO_2_ values that we observed were presumably at least partially caused by the F(SVC)–J(RA) configuration. Secondly, the safety of advancing the drainage cannula into the SVC is not ensured. In this case, as many as four lines passed through the SVC: a drainage cannula, a reinfusion cannula, a central venous catheter and a PAC. Thrombus formation and increased intracranial pressure could have occurred, and there may have been a possibility of vascular perforation by the drainage cannula. Thirdly, the effect of high oxygenated P_PA_O_2_ blood flow passing through the lung is unknown. Since high oxygenated P_PA_O_2_ blood flow cannot pass through the lung physiologically, the F_D_O_2_ was managed with a lower P_PA_O_2_, 0.70 in this case. To avoid the risk of hypoxia it was not lowered below 0.70 because sudden changes might occur, like an increase in recirculation if the cannulae would be unexpectedly repositioned (for example, by the patient’s movements) or an increase in the patient’s cardiac output (CO) resulting in a decrease in the percentage of oxygenated ECMO flow in the RA. Fourthly, the optimal ECMO flow to CO ratio is unknown in the F(SVC)–J(RA) configuration. In normal V-V ECMO management, an ECMO flow to CO ratio of 0.6 or greater must be maintained to keep SaO_2_ above 90% [17]. In the F(SVC)–J(RA) configuration, the optimal ECMO flow to CO ratio may be even lower and may be managed by lowering the ECMO flow instead of lowering F_D_O_2_. Fifthly, the hemodynamics within the right ventricular system, involving how drainage is performed and how the ECMO flow and native venous return flow from SVC and IVC mix and flow into the right ventricle, have not been elucidated. Further analysis, such as flow analysis simulation, is needed. Sixthly, more in general, and this is one of the most important questions, the PaO_2_ advantage may depend on the precise conditions of the patient and the F(SVC)-J(RA) configuration, and we have to study these factors to be able to optimize the technique and predict when it is useful.

## Conclusion

In a V–V ECMO case in which we had advanced the tip of the drainage cannula into the SVC and the reinfusion cannula was crossed in the RA in an “F(SVC)–J(RA) configuration,” a high PaO_2_ far above 200 mmHg was observed despite near-absence of ventilation under lung rest setting. This F(SVC)–J(RA) configuration disagrees with what is conventionally recommended for an F–J configuration, but is very simple by adjusting the tip of the drainage cannula and does not require a special device such as a DLC. We consider the achievement of high systemic oxygenation during V–V ECMO to be revolutionary and believe that it may be very useful in the management of V–V ECMO in patients with severe respiratory failure.

## Supplementary Information

Below is the link to the electronic supplementary material.

Supplementary file1 Supplemental Video 1. Fluoroscopic guidance. The blue bar highlights a drainage cannula, the tip of which was in the superior vena cava. The red bar highlights a reinfusion cannula, the tip of which was in the right atrium. When contrast was injected via the reinfusion cannula, the inferior vena cava became immediately darkly contrasted. (MPEG 11062 KB)

Supplementary file2 Supplemental Video 2. Fluoroscopic guidance. The blue bar highlights a drainage cannula, of which the tip was in the superior vena cava as in Supplemental Video 1. The red bar highlights a reinfusion cannula, of which the tip was pulled upward from its position shown Supplemental Video 1 to the upper right atrium. After injecting contrast via the reinfusion cannula, the right atrium, right ventricle, pulmonary trunk, right pulmonary artery, and left atrium became darkly contrasted in that order. The contrast of the inferior vena cava has faded compared to Supplemental Video 1 (MPEG 11638 KB)

Supplementary file3 Supplemental Video 3. Four chamber view of transesophageal echocardiography focused on the fossa ovalis. The drainage cannula ascending through the right atrium and the tip of the reinfusion cannula in the right atrium are seen. No clear right-to-left shunt flow can be observed upon microbubble test and color Doppler imaging. (MPEG 25130 KB)

## Data Availability

The data that support the findings of this study are available on reasonable request from the corresponding author, TN. The data are not publicly available due to their containing information that could compromise the privacy of the patient.
